# Investing in the use of a checklist during differential diagnoses consideration: what’s the trade-off?

**DOI:** 10.1186/s12909-017-1078-x

**Published:** 2017-11-29

**Authors:** Keng Sheng Chew, Jeroen J. G. van Merrienboer, Steven J. Durning

**Affiliations:** Faculty of Medicine and Health Sciences, Universiti Malaysia Sarawak, Kota Samarahan, Sarawak Malaysia

**Keywords:** Clinical decision making, Diagnostic errors, Cognitive errors, Script concordance test, Checklist, Differential diagnosis

## Abstract

**Background:**

A key challenge clinicians face when considering differential diagnoses is whether the patient data have been adequately collected. Insufficient data may inadvertently lead to premature closure of the diagnostic process. This study aimed to test the hypothesis that the application of a mnemonic checklist helps to stimulate more patient data collection, thus leading to better diagnostic consideration.

**Methods:**

A total of 88 final year medical students were assigned to either an educational intervention group or a control group in a non-equivalent group post-test only design. Participants in the intervention group received a tutorial on the use of a mnemonic checklist aimed to minimize cognitive errors in clinical decision-making. Two weeks later, the participants in both groups were given a script concordance test consisting of 10 cases, with 3 items per case, to assess their clinical decisions when additional data are given in the case scenarios.

**Results:**

The Mann-Whitney U-test performed on the total scores from both groups showed no statistical significance (U = 792, z = −1.408, *p* = 0.159). When comparisons were made for the first half and the second half of the SCT, it was found that participants in the intervention group performed significantly better than participants in the control group in the first half of the test, with median scores of 9.15 (IQR 8.00–10.28) vs. 8.18 (IQR 7.16–9.24) respectively, U = 642.5, z = −2.661, *p* = 0.008. No significant difference was found in the second half of the test, with the median score of 9.58 (IQR 8.90–10.56) vs. 9.81 (IQR 8.83–11.12) for the intervention group and control group respectively (U = 897.5, z = −0.524, *p* = 0.60).

**Conclusion:**

Checklist use in differential diagnoses consideration did show some benefit. However, this benefit seems to have been traded off by the time and effort in using it. More research is needed to determine whether this benefit could be translated into clinical practice after repetitive use.

**Electronic supplementary material:**

The online version of this article (10.1186/s12909-017-1078-x) contains supplementary material, which is available to authorized users.

## Background

Diagnostic error is a pervasive problem that clinicians face irrespective of their years of experience. It is defined as the failure to (a) establish an accurate and timely explanation of the patient’s health problem(s) or (b) communicate that explanation to the patient [1]. One of the largest categories of diagnostic errors is cognitive errors [2]. These are due to one’s predisposition to think in a way that leads to errors in judgment [1, 3]. ‘Search satisficing’ is a form of cognitive error that refers to the tendency of a clinician to stop looking (or to call off a search) for a second diagnosis when the first diagnosis is reached [4]. This can be particularly detrimental in a complex clinical case, such as the inadvertent calling off of a search for associated cervical spine injuries in a patient who sustains a serious head injury.

The process of generating differential diagnoses is said to typically occur in two interrelated steps [5, 6]. The first step typically occurs shortly upon encountering a patient and is called script activation. Scripts are pre-stored structured networks of knowledge that can be activated in working memory in a clinical encounter [5–7]. Script activation is a predominantly non-analytical process whereby one generates possibly relevant diagnoses by linking a cluster of signs and symptoms with his/her previous clinical experiences [5, 6, 8]. As more patient data are collected, additional scripts may be activated. Next, the activated scripts are evaluated on how well they fit the clinical presentation. This stage is known as script evaluation [5, 6]. If deemed less likely with collecting additional data, the existing script may be downplayed or eliminated [5, 6, 9]. This cycle of script activation and script evaluation is believed to be driven by the collection of patient data and is repeated until the point where one decides on the most probable diagnosis that best matches the clusters of signs and symptoms. At this point, the diagnostic process is brought to a closure and the best-matched diagnosis becomes the working diagnosis.

A key challenge that a clinician faces is whether he or she has adequately collected and considered enough patient data, particularly in activating illness scripts that lead to the generation of potentially life- or limb-threatening conditions. Failure to consider these other diagnoses means that the clinician may have prematurely closed the script evaluation process. Checklists aimed to facilitate the script activation process may be helpful in preventing premature closure by stimulating more patient data collection for diagnostic consideration [10, 11].

The script concordance test (SCT) is a tool aimed to assess the participants’ decisions in evaluating the likelihood of a given hypothesis when additional patient data is presented [12]. Each case in SCT is structured with an initial brief clinical vignette that typically contains elements of uncertainty, imprecision or incompleteness. This is then followed by a series of items to evaluate clinical judgment when additional data is provided [12]. Each item has three parts. The first part consists of a hypothesis framed either in the form of a diagnostic possibility, an investigative option or a management option (phrased as “If you are thinking of..”). The second part consists of a new clinical finding that may or may not have an effect on the probability of the hypothesis to be evaluated (phrased “…and then you find…”). The third part is the part where the participants are assessed on, i.e., their evaluation decisions on the likelihood of the given hypothesis based on available additional data measured on a 5-point Likert scale (phrased as “this option would become…”).

With the use of a SCT, this paper aimed to test the hypothesis that the use of a mnemonic checklist, known as the TWED checklist (where the letter ‘T’ = ‘threat’, ‘W’ = ‘what else’, ‘E’ = ‘evidences’ and ‘D’ = ‘dispositional influence’; see Table 1) [13], aids the script evaluation stage by stimulating the consideration of additional relevant patient data, thus, leading to better clinical decisions. The use of this checklist in aiding differential diagnoses generation has been addressed in a previous study [11].

**Table 1:** The TWED Checklist

T = life or limb Threat	W = Wrong?
(What are the life or limb threatening conditions in this patient?)	(What if I am wrong? What else could it be?)
This quadrant encapsulates the rule-out-worse-case scenarios (ROWS) heuristics as a form of cognitive forcing strategy as well as to de-bias anchoring and triage cueing	To de-bias search satisficing, anchoring, confirmation, availability biases, etc
E = Evidences	D = Dispositional factors
(Do I have sufficient evidences for or exclude this diagnose?)	(What are the Environmental & Emotional (2Es) dispositions influencingmy decision?)
To de-bias anchoring, confirmation bias, blind spot, myside bias, ego bias, etc	These dispositional factors that may affect our decision making. Examples: Environmental – chaotic, busy working place, Emotional – sleepiness, tiredness, anger

## Methods

### Participants

A total of 88 final year (Year 5 cohort 2014/2015) medical students from Universiti Sains Malaysia (USM) (mean age = 23.20, SD = 0.42 years; 44% male) participated in this anonymous, voluntary study using a non-equivalent control group, posttest-only design. The undergraduate medical program in USM is a five-year program, where the first 3 years are considered as the pre-clinical years and Years 4 and 5 are the clinical years (although students in Years 2 and 3 have some clinical exposure in history taking and physical examination). To qualify for Year 5 clinical clerkship, all these students had satisfactorily passed the examinations in Year 4 clerkship including internal medicine, pediatrics, general surgery, obstetrics, gynecology and psychiatry. All students go through their clinical clerkship in different disciplines on a rotational basis in groups of 20–30 students per clinical group. Two out of four clinical groups were randomly assigned to receive educational intervention (*N* = 48, mean age = 23.17, SD = 0.37 years; 45.8% male) and another two clinical groups were assigned as the control (*N* = 40, mean age = 23.22, SD = 0.48 years; 42.5% male). Participants in the control group did not receive any educational intervention on the use of the TWED checklist.

### Materials

The educational intervention received by participants in the intervention group consisted of a 2-h tutorial on factors contributing to making diagnostic errors, strategies to minimize the risk of committing cognitive errors, including the application of the TWED mnemonic checklist in clinical cases (hence, the group is named, the ‘TWED group’). This tutorial was given in a classroom setting of about 20–30 participants. A hand-out of the tutorial was also given to the participants during the class.

The TWED mnemonic checklist is a recently developed checklist aimed to minimize cognitive errors [11, 13]. In particular, the quadrants ‘T’, ‘W’ and ‘E’ may be helpful in stimulating the collection and consideration of more patient data whereas quadrant ‘D’ acts as an overarching self-reflective mechanism to guard against premature closure due to extrinsic influences. As an example, suppose the participant is given this initial brief clinical scenario: “A 45-year old man complains of chest pain and shortness of breath after a blunt trauma to the chest”. Immediately, the participant is likely to activate pneumothorax and/or myocardial contusion scripts. In a question that follows this brief scenario, the participant is given a conditional statement in two parts: “If you were thinking of ordering an electrocardiography (ECG)”, “and then you were to find lower right-sided chest tenderness on palpation”. He or she is then asked to evaluate the usefulness of ECG on a 5-point Likert scale. By reflecting on the first and second quadrant of the TWED checklist (“T = threat” and “W = what else”), the additional data of “right-sided lower chest tenderness on palpation” may stimulate the activation of the additional illness script of rib fracture. Whereas reflecting on the third quadrant (“E = evidence”) may trigger the consideration of how well this additional data of “right-sided lower chest tenderness on palpation” fits as an evidence for the existing scripts of pneumothorax and myocardial contusion. The hypothesis is that these considerations using the checklist could strengthen the diagnoses of pneumothorax with the associated diagnosis of rib fractures, and lead to better decision making for evaluating how useful an ECG would be.

As previously stated, an SCT test (consisting of 10 cases, with 3 items per case) was used to assess the participant’s decisions on the likelihood of a given hypothesis when additional patient data are given. Based on the principles of SCT development [12, 14], each of these cases was constructed with a short clinical vignette followed by three items with three parts for each item. An example of the components of an SCT case (the first case of these 10 cases) is given in Table 2. The participants’ responses to this part will be scored based on the scoring key described below.

**Table 2:** Example of a case constructed with 3 items, with 3 parts for each item in script concordance test

Case 1						
A 45-year old man complains of chest pain and shortness of breath after a blunt trauma to the chest is brought to the emergency department.						
If you were thinking of	…and then you were to find	…you would then consider this action −2 completely or almost completely unnecessary −1 less useful 0 neither more nor less useful +1 useful +2 completely or almost completely necessary				
Ordering an electrocardiogram (ECG)	Lower right-sided chest tenderness on palpation	−2	−1	0	+1	+2
Ordering a computed tomography of the brain (CT brain)	No history of loss of consciousness	−2	−1	0	+1	+2
Ordering an abdominal radiograph	Soft and non-tender abdomen on palpation	−2	−1	0	+1	+2

The ten SCT cases were developed by one of the authors (KSC) and were independently reviewed by two emergency physicians to ascertain that the cases in this test were a reasonable sample of frequently occurring cases that future house officers commonly encounter. The two emergency physicians also evaluated the comprehensibility of these cases including their readability as well as grammatical correctness. Feedback received was incorporated in the revised version of the cases. The detailed descriptions of the 10 cases are provided as Additional file 1.

Ten emergency physicians from different hospitals and institutions in Malaysia were invited to become the panel members in the development of the scoring key for the cases and all of them agreed. All panel members had at least 15 years of experience as clinicians and at least 10 years in the field of emergency medicine. To account for the variability of experts’ responses to a particular clinical situation, the recommended aggregate scoring method [12, 14] was adopted. In this aggregate scoring method, although the answer provided by the greatest number of panel members is assumed to be the optimal decision under the given situation, other answers provided by other panel members reflect a difference of interpretation that still merits proportional credit [12, 14]. For example, in one of the items, nine out of 10 experts chose the response “-1” and one of them chose “-2”. The response selected by most experts or the modal response (in this case, “-1”) is credited with the maximum credit of 1 mark. Other responses are given partial credit in proportion to the number of experts who selected that particular response divided by the modal (in this example, the response “-2” is accorded 1/9 or 0.18 point). Each of these three items is given a maximum credit of 1 point and as there are three items per case, there would be a total of 3 points per case. Thus, the maximum score of each case is 3 points and the maximum score of the whole SCT is 30 points. A worked example as described above is shown in Table 3.

**Table 3:** Example of how the scoring key is developed based on the aggregate scoring method in script concordance test

Response	−2	−1	0	+1	+2
Number of experts who choose this response	9	1	0	0	0
Transformed score	1/9	9/9	0	0	0

### Procedure

The educational intervention for the TWED group was implemented at the beginning of the participants’ 2-week emergency medicine rotation. The participants were told to use the checklist as often as they could during their 2 weeks’ rotation. Two weeks later, the participants were asked to independently complete the paper-based SCT in a classroom setting. They were also told to use on the TWED checklist when answering the SCT. Students in the control group were similarly asked to complete these same 10 cases. These students were not exposed to the educational intervention of using the TWED checklist. After completion of the SCT, a general feedback was obtained on the participants’ perception on applying the TWED checklist during the SCT.

Based on the guideline by Fournier et al. (2008) [14], all participants were given 30 min to complete the test. Ethics approval from the institutional ethics and research committee of Universiti Sains Malaysia was granted for this study.

## Results

The Mann-Whitney U-test was used to analyze the total score differences between the two groups as the normality of data cannot be assumed in the intervention group with the Shapiro-Wilk test (skewness z-value = 2.86, and kurtosis z-value = 3.53, *p* = .04). Homogeneity of variances in the sample was verified by using the non-parametric Levene’s test (*p* = 0.33).

The inter-rater reliability of the 10 panel members, determined using two-way mixed effect model of intra-class correlation coefficient (ICC), was found to be 0.93 (95% CI 0.89–0.96). The Cronbach’s alpha for the internal consistency of the test was 0.93, which is very good.

All participants from both groups completed the SCT consisting of 10 cases (with 3 items per case) within the stipulated time of 30 min. None of the questions were left unanswered. The median total scores for the TWED group and control group were 18.65 (inter-quartile range, IQR, of 16.96–20.34) and 18.15 (IQR 16.79 to 19.37) out of 30, respectively, U = 792, z = −1.408, *p* = 0.159. Thus, no significant difference between groups was found.

However, as the consideration of more patient data resulting from the use of the TWED checklist might possibly have been a time-consuming effort affecting the performance of the participants in the TWED group, a comparison between the TWED group and the control group was made for the first half and the second half of the SCT. It was found that in the first half of the SCT, participants in the TWED group outperformed participants in the control group. But no similar difference was noted in the second half of the SCT. A Mann-Whitney test performed on the test scores for the first half of the test (first 5 cases) showed that the median score of those in the TWED group was 9.15 over a total of 15 marks (IQR 8.00–10.28), and this is significantly higher than the median score in the control group, 8.18 (IQR 7.16–9.24), U = 642.5, z = −2.661, *p* = 0.008. This is not the case with the second half of the test, where the median scores in the intervention group and control group were 9.58 (IQR 8.90–10.56) and 9.81 (IQR 8.83–11.12), respectively, U = 897.5, z = −0.524, *p* = 0.60 (see Table 4).

**Table 4:** Results of the SCT score in the TWED group vs control group

	TWED group		Control group	
	Median	IQR	Median	IQR
First half of test	9.15	8.00–10.28	8.18	7.16–9.24
Second half of test	9.58	8.90–10.56	9.81	8.83–11.12
Total test score	18.65	199.96–20.34	18.15	16.79–19.37

With regards to the feedback on the use of the checklist among participants in the TWED group, although most of these participants felt that the SCT was not too difficult to perform, a number of them felt that the duration of 30 min was too short for them to thoroughly apply the TWED checklist in all cases. A few of them commented that they had apparently spent too much time on the initial few cases so that they had to rush through the rest of the cases. This seems to be in keeping with the observations from the statistical results that the TWED group outperformed the control group in the initial few cases, but not for the subsequent cases because of the trade-off between using the checklist and time pressure with completing the test.

## Discussion

The results of this study partly support the hypothesis that the use of a checklist aids the script evaluation stage. The checklist appeared to benefit the TWED group by acting as a self-regulatory prompt for a more careful consideration of the additional patient data, which in turn, may have resulted in the activation of additional illness scripts. This minimizes the risk of committing premature closure but also seems to take more time. This is in keeping with the observation that participants in the TWED group outperformed those in the control group for the first half of the test scores, but not for the second half.

Thus, it seems that the participants in the TWED group applied the checklist in the first half of the SCT and hence attained better scores, but as the application of the TWED checklist was a relatively new task for these participants, it was a time-consuming effort. In a time-pressured setting (such as in this study), they might have had to abandon the use of the checklist in the second half of the test in order to complete all items in time. This is consistent with the comments given by some of the participants in the TWED group who felt that the 30 min given to them were not adequate to complete the full test comfortably.

This inconsistency between test results for the first half and the second half of the SCT could be explained by the postulation that while the application of the TWED checklist might have benefited the participants if given as much time as needed, this checklist may have also imposed a heavier cognitive load on these participants compared to participants who were not given the checklist (the control group). A higher cognitive load imposed by the task typically leads to the need to invest more mental effort and/or more time to accommodate the task demands [15, 16].

Undoubtedly, considering differential diagnoses is a complex cognitive task with a high level of element interactivity. Element interactivity refers to the necessity for multiple items to be held and processed simultaneously in working memory [16]. Learning the names of the different muscles in the upper limb has a low level of element interactivity as each of these muscles can be learned serially and independently from one another. The consideration of differential diagnoses, in contrast, imposes a high level of element interactivity as the clusters of signs and symptoms need to be held simultaneously in working memory for consideration. While it is essential for pre-stored items such as illness scripts to be activated into working memory, the capacity of working memory is severely limited [7, 16, 17]. Miller (1956), for example, in his classic paper [17] stated that working memory is capable of holding about 7 (plus or minus 2) items at a given time. In other words, the advantage of using this relatively new checklist was traded off with the additional cognitive load imposed.

Future studies should look at the effect of the TWED checklist after the participants have repeatedly used it in practice over a long period of time. Repetitive practice will allow for more illness scripts construction and assimilation of the checklist [7, 16]. In this regard, it is likely that through repetitive practice, dealing with the questions posed in the TWED checklist can eventually be embedded seamlessly in differential diagnoses consideration.

Studies could also be done to look at the differential effects of the checklist on the various types of cognitive load. Cognitive load is a multi-dimensional concept describing the load imposed on the cognitive system due to a task performance [15, 16]. Sweller et al. (1998) [16] described three distinct types of cognitive loads, i.e., intrinsic, extraneous as well as germane cognitive loads. While intrinsic load is an inherent function of performing a task and cannot be altered by changing the instructional design, extraneous load is the unnecessary load imposed due to a poorly designed instruction. Germane load, on the other hand, refers to the load that directly contributes to the construction of more illness scripts as well as task automation so long as the total cognitive load (intrinsic plus extraneous plus germane cognitive load) stays within the limitation of the working memory. The brevity and the mnemonic structure of the TWED checklist could have minimized the total cognitive load to ensure that it stays within the limitation of the working memory, while the yielded germane load of its application allows for the assimilation of the checklist after repetitive practices.

This study has a number of limitations. Although participants in both groups were medical students who had successfully passed the examination required to qualify them for final year study, it was conducted as a non-equivalent, post-test only design without a similar pre-test. Although student cohorts have identical backgrounds and were randomly assigned to the experimental groups, there is still the possibility that participants in the two groups might have differed in terms of the depth of their prior knowledge and experience. This might have had a confounding effect on their test performance. Secondly, as this study was conducted in a classroom setting, it lacks the ecological validity of a real clinical environment. Thirdly, this study entails testing the use of checklist on a single occasion, which may not reveal the potential long-term effects. Fourthly, unlike the participants, although the expert panel members were told of the guideline [14] where each item is allocated 1 min for completion, no strict time limit was imposed on the expert panels to complete their responses. Should the panel members be given a similar time limit of 30 min to complete their responses, this might have produced a scoring key that is more reflective of expert responses in a time-pressured setting. Besides, there have been some recent concerns with regards to the validity of the recommended aggregate scoring method in SCT [18] as this method incorporates non-modal responses, even if these non-modal responses fall on the opposite side of the Likert scale from the modal responses. Nonetheless, as participants from both groups were given exactly the same set of SCT, this limitation is more of an inherent limitation of the construct of the SCT per se. Finally, this study only tested the effect of the TWED checklist on a single occasion. Hence, the results of this study may not truly reflect the long-term effect of the checklist on differential diagnoses consideration.

In summary, albeit its limitations, this study suggests that checklist use in differential diagnoses consideration does have some beneficial effect. But even then, our findings suggest that the clinician needs to “get use to” the checklist. It is only after considerable practice that one can expect to reap this benefit without trading off the time and effort invested in using it.

## Conclusion

Diagnostic error is a pervasive problem in clinical practice due to a number of causes including premature closure during the process of differential diagnoses consideration. This study partly supports the hypothesis that the TWED checklist is useful in minimizing the risk of premature closure by stimulating more patient data collection and consideration. Future works are needed to study the long-term effect of using checklists in differential diagnoses consideration.

## Additional file

Additional file 1: Descriptions of the ten case scenarios in script concordance test. (DOC 89 kb)

## Abbreviations

SCT: Script concordance test. It is an assessment tool aimed to assess clinical decisions on the likelihood of a given hypothesis when additional patient data is presented
TWED: Is a mnemonic checklist aimed to minimize the risk of committing cognitive errors. The letter ‘T’ stands for ‘threat’, ‘W’ = ‘what else’, ‘E’ = ‘evidences’ and ‘D’ = ‘dispositional factors’, which can be further divided into 2 Es, namely, Environmental & Emotional factors
USM: Universiti Sains Malaysia
