# Supplementary material for: Investing in the use of a checklist during differential diagnoses consideration: what’s the trade-off?
Source: BMC Med Educ. 2017 Nov 29;17:234. doi: 10.1186/s12909-017-1078-x (PMC5707798; doi:10.1186/s12909-017-1078-x)
Supplement: Additional file 1: — Descriptions of the ten case scenarios in script concordance test. (DOC 89 kb) [file 12909_2017_1078_MOESM1_ESM.doc]

**Script Concordance Testing**

Case 1

A 45-year old man complains of chest pain and shortness of breath after a blunt trauma to the chest is brought to the emergency department.

| If you were thinking of | …and then you were to find | …you would then consider this action  -2 not useful at all  -1 less useful  0 neither more nor less useful  +1 useful  +2 very useful |
| --- | --- | --- |
| Ordering an electrocardiogram (ECG) | Lower right-sided chest tenderness on palpation | | -2 | -1 | 0 | +1 | +2 | | --- | --- | --- | --- | --- | |
| Ordering a computed tomography of the brain (CT brain) | No history of loss of consciousness | | -2 | -1 | 0 | +1 | +2 | | --- | --- | --- | --- | --- | |
| Ordering an abdominal radiograph | Soft and non-tender abdomen on palpation | | -2 | -1 | 0 | +1 | +2 | | --- | --- | --- | --- | --- | |

Case 2

A healthy 30-year old lady is brought to the emergency department by the ambulance after fainting at home. On arrival to the house, the ambulance crew finds that she is awake, oriented with a GCS of 15/15.

| If you were thinking of | …and then you were to find | …you would then consider this action  -2 not useful at all  -1 less useful  0 neither more nor less useful  +1 useful  +2 very useful |
| --- | --- | --- |
| Performing a electrocardiogram (ECG) | The patient has no history of chest pain | | -2 | -1 | 0 | +1 | +2 | | --- | --- | --- | --- | --- | |
| Ordering a urine pregnancy test | The patient also complains of right shoulder tip pain | | -2 | -1 | 0 | +1 | +2 | | --- | --- | --- | --- | --- | |
| Ordering a computed tomography | The family witnesses describe her fainting as brief loss of consciousness lasting around 30 – 60 seconds | | -2 | -1 | 0 | +1 | +2 | | --- | --- | --- | --- | --- | |

Case 3

A 12-month old girl is brought to the emergency department with a 3-day history of sleepiness, multiple episodes of vomiting and reduced feeding. On examination, her temperature is 37.5°C, BP 90/40 mmHg, pulse rate 90/min; her mucous membranes are dry and capillary refill is 4 seconds.

| If you were thinking of | …and then you were to find | …you would then consider this action  -2 not useful at all  -1 less useful  0 neither more nor less useful  +1 useful  +2 very useful |
| --- | --- | --- |
| Ordering a serum electrolytes and blood urea level | Her vomiting is non-bilious | | -2 | -1 | 0 | +1 | +2 | | --- | --- | --- | --- | --- | |
| Ordering a computed tomography (CT) of the brain | Her BP is 100/50 mmHg and pulse rate 80/min after two boluses (20 ml/kg) of IV Ringer’s lactate for fluid resuscitation | | -2 | -1 | 0 | +1 | +2 | | --- | --- | --- | --- | --- | |
| Ordering a barium enema | Vague mass at right upper quadrant on abdominal palpation | | -2 | -1 | 0 | +1 | +2 | | --- | --- | --- | --- | --- | |

Case 4

A 23-year-old lady is admitted to the emergency department with two episodes of generalized tonic-clonic seizures. This patient has fever of 38.5°C

| If you were thinking of | …and then you were to find | …you would then consider this diagnosis  -2 very unlikely  -1 unlikely  0 neither likely nor unlikely  +1 likely  +2 very likely |
| --- | --- | --- |
| Cerebral abscess | The patient has been hearing voices talking about her | | -2 | -1 | 0 | +1 | +2 | | --- | --- | --- | --- | --- | |
| Connective tissue disorder | The patient has complaints of joint pain involving small joints of hands | | -2 | -1 | 0 | +1 | +2 | | --- | --- | --- | --- | --- | |
| Malaria | The patient has generalized lymphadenopathy | | -2 | -1 | 0 | +1 | +2 | | --- | --- | --- | --- | --- | |

Case 5

A 34-year old foreign man comes to the emergency department with a complaint of acute chest pain. You have difficulty in communicating with him due to language barrier.

| If you were thinking of | …and then you were to find | …you would then consider this action  -2 not useful at all  -1 less useful  0 neither more nor less useful  +1 useful  +2 very useful |
| --- | --- | --- |
| 1. Ordering a chest radiograph (chest X-ray) | Generalized bilateral crepitations on lung auscultation | | -2 | -1 | 0 | +1 | +2 | | --- | --- | --- | --- | --- | |
| 2. Ordering thyroid function test (TFT) | Irregularly irregular pulse rate | | -2 | -1 | 0 | +1 | +2 | | --- | --- | --- | --- | --- | |
| 3. Performing an echocardiography | The patient has a history of upper respiratory tract infection | | -2 | -1 | 0 | +1 | +2 | | --- | --- | --- | --- | --- | |

Case 6

A 50-year-old lady complains of generalized body weakness.

| If you were thinking of | …and then you were to find | …you would then consider this diagnosis  -2 very unlikely  -1 unlikely  0 neither likely nor unlikely  +1 likely  +2 very likely |
| --- | --- | --- |
| Guillain-Barré syndrome | The weakness has been progressive over the last 24 hours | | -2 | -1 | 0 | +1 | +2 | | --- | --- | --- | --- | --- | |
| Myastenia gravis | The patient has difficulty opening her eyes, especially in the evenings | | -2 | -1 | 0 | +1 | +2 | | --- | --- | --- | --- | --- | |
| Hyperkalemia | Profound weakness in the proximal muscles of both upper and lower limbs | | -2 | -1 | 0 | +1 | +2 | | --- | --- | --- | --- | --- | |

Case 7

A 20-year old college girl is found drowsy in her hostel room. According to her roommate, she last saw her yesterday evening when she went out with her boyfriend. On examination, her GCS is 9/15. No neck stiffness.

| If you were thinking of | …and then you were to find | …you would then consider this action  -2 not useful at all  -1 less useful  0 neither more nor less useful  +1 useful  +2 very useful |
| --- | --- | --- |
| 1. Ordering a urine pregnancy test | Her roommate says that her last menses was about 3 weeks’ ago. | | -2 | -1 | 0 | +1 | +2 | | --- | --- | --- | --- | --- | |
| 2. Ordering a computed tomography of the brain (CT brain) | Her pupils were responsive to light with size 3 mm bilaterally. | | -2 | -1 | 0 | +1 | +2 | | --- | --- | --- | --- | --- | |
| 3. Ordering a serum paracetamol level | there was an empty bottle with the label “PARATECETAMOL”. | | -2 | -1 | 0 | +1 | +2 | | --- | --- | --- | --- | --- | |

Case 8

A 79-year old lady complains of progressive dyspnea over several hours.

| If you were thinking of | …and then you were to find | …you would then consider this action  -2 not useful at all  -1 less useful  0 neither more nor less useful  +1 useful  +2 very useful |
| --- | --- | --- |
| Ordering of D-dimer | The patient is immobile | | -2 | -1 | 0 | +1 | +2 | | --- | --- | --- | --- | --- | |
| Ordering an electrocardiograph (ECG) | The patient denies any history of chest pain | | -2 | -1 | 0 | +1 | +2 | | --- | --- | --- | --- | --- | |
| Performing a full blood count | Scattered bilateral rhonchi on auscultation | | -2 | -1 | 0 | +1 | +2 | | --- | --- | --- | --- | --- | |

Case 9

A 50-year old man complains of severe throbbing headache over the last 4 hours which has never happened to him before.

| If you were thinking of | …and then you were to find | …you would then consider this diagnosis  -2 very unlikely  -1 unlikely  0 neither likely nor unlikely  +1 likely  +2 very likely |
| --- | --- | --- |
| Subarachnoid hemorrhage | the presence of neck stiffness | | -2 | -1 | 0 | +1 | +2 | | --- | --- | --- | --- | --- | |
| Pituitary tumor | Visual impairment | | -2 | -1 | 0 | +1 | +2 | | --- | --- | --- | --- | --- | |
| Migraine | Photophobia | | -2 | -1 | 0 | +1 | +2 | | --- | --- | --- | --- | --- | |

Case 10

A 40-year old man accidentally fell from a height of 12 meter. His vital signs are as follow: BP 70/50 mmHg, SaO2 91% under non-rebreathing high flow oxygen, respiratory rate 30 breaths/min, GCS 9/15.

| If you were thinking of | …and then you were to find | …you would then consider this action  -2 not useful at all  -1 less useful  0 neither more nor less useful  +1 useful  +2 very useful |
| --- | --- | --- |
| Immediate endotracheal intubation with in-line stabilization of cervical spine | Gurgling sound heard with each attempted breath | | -2 | -1 | 0 | +1 | +2 | | --- | --- | --- | --- | --- | |
| Immediate chest tube insertion | Diminished breath sounds on right lung upon auscultation | | -2 | -1 | 0 | +1 | +2 | | --- | --- | --- | --- | --- | |
| Immediate splinting | His right leg is grossly deformed | | -2 | -1 | 0 | +1 | +2 | | --- | --- | --- | --- | --- | |
